# Feasibility and diagnostic performance of dual-tracer-guided sentinel lymph node biopsy in cT1-2N0M0 gastric cancer: a systematic review and meta-analysis of diagnostic studies

**DOI:** 10.1186/s12957-017-1159-7

**Published:** 2017-05-16

**Authors:** Ling Huang, Tao Wei, Junjun Chen, Donghui Zhou

**Affiliations:** 1 Department of Oncological Surgery, The First Affiliated Hospital of College of Medicine of Zhejiang University, Qingchun Road 79, Hangzhou, Zhejiang Province China; 2 Department of Respiratory Diseases, The First Affiliated Hospital of College of Medicine of Zhejiang University, Qingchun Road 79, Hangzhou, Zhejiang Province China

**Keywords:** Dual tracer, Stomach neoplasm, Sentinel lymph node

## Abstract

**Background:**

Dual-tracer-guided sentinel lymph node (SLN) biopsy may provide a promising diagnostic tool to assess accurately the status of lymph node metastasis in the surgical operation and assure the oncologic safety of the function or stomach preserving surgery. The diagnostic performance of this technology in recent studies varied. Thus, we conducted this meta-analysis.

**Methods:**

This systematic review and meta-analysis was registered at the PROSPERO. Eligible studies were searched in the PubMed, EMBASE, Web of Knowledge, and Cochrane Library databases. A random-effect model was used to pool the data. Summary receiver operator characteristic curves, analysis for publication bias, meta-regression, and subgroup analysis were also performed.

**Results:**

The pooled SLN identification rate and sensitivity were 0.97 and 0.89. ^99m^Tc-human serum albumin with indocyanine green (ICG), ^99m^Tc-antimony sulfur colloid with ICG, performing SLN biopsy ≥15 min after dye injection, an SLN ≥5, the basin dissection, laparoscopic surgery, in studies conducted in Japan and studies published after 2012, were associated with higher sensitivity. CT1 stage, performing SLN biopsy ≥15 min after dye injection, in studies conducted in Japan and studies published after 2012, were related with a higher identification rate.

**Conclusions:**

Dual tracer is promising in SLN biopsy in gastric cancer, and the clinical application of SLN biopsy should be limited to the patients of cT1N0M0 gastric cancer. The combination of ^99m^Tc-human serum albumin and ICG as well as the combination of ^99m^Tc-antimony sulfur colloid and ICG may be the optimal tracer combination. However, it seems not justified to put this technique into routine clinical application recently. Some factors that might enhance diagnostic value are identified.

**Electronic supplementary material:**

The online version of this article (doi:10.1186/s12957-017-1159-7) contains supplementary material, which is available to authorized users.

## Background

More and more early gastric cancer has been diagnosed recently due to the advances in screening methods such as endoscopic examination [1, 2]. According to the Gastric Cancer Treatment Guidelines 2014 of Japanese Gastric Cancer Association, a D1 lymphadenectomy or D1+ lymphadenectomy is indicated for cT1N0M0 gastric cancers. Since the preoperative diagnosis of lymph node metastasis remains unreliable, a D2 lymphadenectomy is recommended whenever lymph node metastasis is suspected and the prophylactic lymphadenectomy may lead to the postoperative complications such as chylous ascites [3, 4]. Besides, segmental gastrectomy and local resection are now on trial in cT1N0M0 gastric cancers. These kinds of function or stomach preserving surgery can avoid the complications of traditional total or distal gastrectomy such as dumping syndrome and malnutrition [5]. Thus, a diagnostic tool is needed to assess accurately the status of lymph node metastasis in the surgical operation and assure the oncologic safety of the function or stomach preserving surgery [6–8].

Sentinel lymph node (SLN) biopsy may provide a promising tool to resolve this issue. The SLNs are defined as the first possible lymph nodes to which the primary tumor drains. They are detected with the help of tracers and then resected for intraoperative pathologic examination. And the pathologic results of SLNs are believed to predict the lymphatic metastatic status of downstream lymph nodes [9]. However, lymphatic flow of the stomach is complicated and there is the possibility of skip metastasis [10]. Thus, the application of SLN biopsy in gastric cancer has long been debated [11]. In addition, suitable tracers are still controversial in SLN detection in gastric cancer. At present, the radioisotope and dye are commonly used as tracers, each of which has its own merits and shortcomings in SLN detection. So, some researchers have adopted the combination of these two tracers. Recently, several studies about dual-tracer-guided SLN biopsy in gastric cancer have been carried out; however, the diagnostic performance and procedures of SLN biopsy in these studies varied. In this meta-analysis, we attempt to evaluate the feasibility and diagnostic performance of dual-tracer-guided SLN biopsy in cT1-2N0M0 gastric cancer. To confirm the proper indications of SLN biopsy in terms of the depth of the primary tumor, the studies with patients of cT1N0M0 or cT2N0M0 gastric cancer are all involved in our meta-analysis. Furthermore, the secondary goals are to identify factors that may enhance diagnostic value.

## Methods

This systematic review and meta-analysis was registered at the PROSPERO. The registration number is CRD42016046730.

### Literature search strategy

The electronic databases PubMed, EMBASE, Web of Knowledge, and the Cochrane Library were searched from initiation of the databases to September 3, 2016. Free text and medical subject heading terms were used for stomach neoplasm and sentinel lymph nodes. The language was not limited. To search for additional potentially relevant articles, reference lists from the included trials were screened. Furthermore, to obtain any relevant full texts and missing data, we contacted the authors of the papers.

### Inclusion and exclusion criteria

The inclusion criteria for this meta-analysis were as follows: (a) studies assessed the diagnostic value of dual-tracer-guided SLN biopsy in predicting the lymph node status of cT1-2N0M0 gastric cancer and (b) the sample size was greater than 10 patients.

Studies were excluded based on the following criteria:(a) reviews, case reports, meta-analyses, abstracts, or letters; (b) in vitro studies and studies performed on animals; and (c) studies without sufficient data of diagnostic performance.

Study search and selection were performed independently by two investigators (L.H. and T.W.). Disagreements between the two investigators were resolved by a third investigator (J-J.C.) after re-checking the original article and discussion of the evidence.

### Data extraction and quality assessment

Two authors (L.H. and T.W.) extracted data using predefined tables. The results from patients with successfully identified SLNs were recorded as TP, FN, or TN. TP was defined as the number of patients whose SLNs were positive with or without positive non-SLNs. FN was defined as the number of patients whose SLNs were negative with the positive non-SLNs. TN was defined as the number of patients whose SLNs were negative with negative non-SLNs.

QUADAS-2 was used in our review to assess risk of bias and applicability concerns [12]. All of the studies were independently assessed by two reviewers (L.H. and J-J.C.), and any disagreement was resolved by discussion.

### Statistical analysis

Statistical analyses were performed in Meta-DiSc software Version 1.4 (Javier Zamora, Madrid, Spain), Comprehensive Meta Analysis software Version 3.0 (Biostat Inc., Englewood, NJ, USA), and STATA software Version 12.0 (Stata Corporation, TX, USA). All the statistical analysis were reviewed and confirmed by J-J.C. whose second major is statistics.

Random-effects models for the meta-analysis were used to calculate pooled proportions for identification rates, sensitivities (TP/(TP + FN)) and negative likelihood ratios (LR) ((1 − sensitivity)/specificity) with 95% confidence interval (CI). Spearman’s correlation coefficient was used to detect the threshold effect. Forest plots showed variations in the study results and pooled estimates. The summary receiver operator characteristic (SROC) curve showed diagnostic accuracy by the area under the curve (AUC) and the Q* index (a statistical value defined by the point on the SROC curve where sensitivity and specificity are equal) [13].

Statistical heterogeneity was tested with a chi-squared test and was quantified by *P* value. Statistical heterogeneity was considered when *P* <0.1. Publication bias was evaluated by funnel plots and by the Egger’s test for funnel plot asymmetry.

If heterogeneities were present, stratum-specific pooled estimates were generated for subgroup analysis. Meta-regression analyses were also performed to find factors determining the diagnostic accuracy if sufficient studies were available. *P* < 0.05 was considered statistically significant.

## Results

### Study selection

**Fig. 1:**
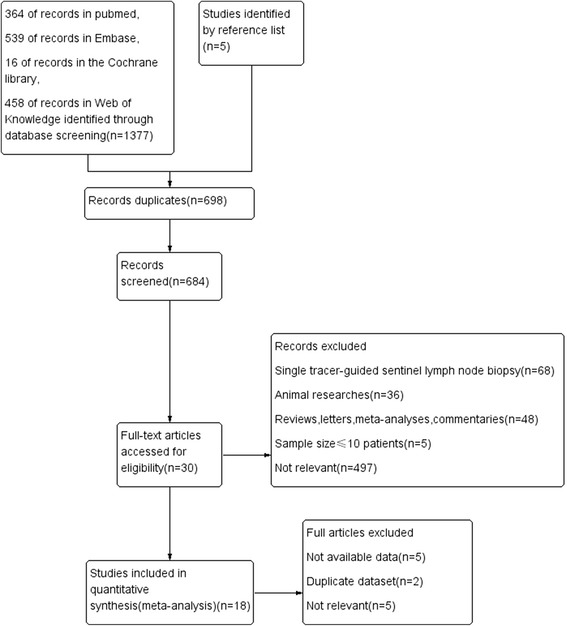
Flow diagram for study selection

Eighteen feasibility studies involving 1663 patients were enrolled in this meta-analysis. Of the included studies, one study analyzed the data separately according to different biopsy methods (basin dissection vs pick up). The study selection process is summarized in Fig. 1.

### The quality of the literature studies

The results of the quality assessment are listed in Table 1 [14–31].

**Table 1:** The results of quality assessment according to QUADAS 2 for the included studies

Study id	Risk of bias				Applicability concerns		
	Patient selection	Index test	Reference standard	Flow and timing	Patient selection	Index test	Reference standard
Ayako Shimada2016	1	1	1	1	1	1	1
Masahiro Niihara2016	1	1	1	1	1	1	1
Young Joon Lee2015	1	1	1	1	1	1	1
Satoshi Kamiya2015	1	1	1	1	1	1	1
Shuhei Mayanagi2014	2	2	?	?	1	?	?
Yuko Kitagawa2013	1	1	1	1	1	1	1
Ju-Hee Lee2013	1	1	?	?	1	1	?
Do Joong Park2011	1	1	1	1	1	1	1
Hiroya Takeuchi2011	1	2	?	?	1	?	?
Takashi Ichikura2009	1	1	1	1	1	1	1
Jun Ho Lee 3^rd^ trial 2009	1	2	1	1	1	1	1
Young-Joon Lee2008 pick up	1	1	?	2	1	1	?
Young-Joon Lee2008 basin	1	1	?	?	1	1	?
Jun Ho Lee2006	1	1	1	1	1	1	1
Yoshiro Saikawa2006	1	2	?	?	1	1	?
Hitoshi Tonouchi2005	1	1	1	1	1	1	1
Tomoaki Karube2004	1	1	1	1	1	1	1
Hideki Hayashi2003	1	1	1	1	1	1	1
Hitoshi Tonouchi2003	2	1	1	1	1	1	1

*1* low, *2* high, *?* unclear

### General study characteristics

The studies involved two multicenter prospective clinical studies with sample sizes varying from 17 to 397. The tumor size of most patients was <4 cm. The characteristics of the included studies are listed in Table 2 and Additional file 1: Table S1 and Table S2.

**Table 2:** Patient characteristics of included studies

Author	Year	Country	Sample size	Patient age	Preoperative tumor stage	Tumor size (cm)	L/O
Ayako Shimada et al.	2016	Japan	156	59.5 ± 11.5	CT1N0M0	<4 cm	Both
Masahiro Niihara et al.	2016	Japan	385	26–86	CT1-2aN0M0	<4.0 cm in 286 patients, >4.0 cm in 99 patients	Both
Young Joon Lee et al.	2015	South Korea	108	20–80	CT1-2N0M0	<4 cm	L
Satoshi Kamiya et al.	2015	Japan	72	61.5 ± 12.7	CT1N0M0	<4 cm	O
Shuhei Mayanagi et al.	2014	Japan	40	66.0 ± 9.1	CT1N0M0	<4 cm	O
Yuko Kitagaw et al.	2013	Japan	397	29–87	CT1-2N0M0	<4 cm	O
Ju-Hee Lee et al.	2013	South Korea	24	40–80	CT1N0M0	≤4 cm	L
Do Joong Park et al.	2011	South Korea	68	55.9 ± 10.7	CT1-2N0M0	<4 cm	L
Hiroya Takeuchi et al.	2011	Japan	37	62.0 ± 12.0	CT1N0M0	<4 cm	L
Takashi Ichikura et al.	2009	Japan	38	30–81	CT1N0M0	<4 cm	O
Jun Ho Lee 3rd trial et al.	2009	South Korea	21	28–76	CT1N0M0	2–4 cm	L
Young Joon Lee (pick-up) et al	2008	South Korea	42	30–81	CT1-2N0M0	≤4 cm	L
Young Joon Lee (basin) et al.	2008	South Korea	50	35–85	CT1-2N0M0	≤4 cm	L
Jun Ho Lee et al.	2006	South Korea	64	60.0 ± 11.0	CT1N0M0	<5 cm	O
Yoshiro Saikawa et al.	2006	Japan	35	41–77	CT1N0M0	Unclear	L
Hitoshi Tonouchi et al.	2005	Japan	37	Unclear	CT1-2N0M0	Unclear	L
Tomoaki Karube et al.	2004	Japan	41	59 ± 10	CT1-2N0M0	Unclear	O
Hideki Hayashi et al.	2003	Japan	31	42–77	CT1-2N0M0	Unclear	O
Hitoshi Tonouchi et al.	2003	Japan	17	52–85	CT1N0M0	Unclear	L

*L* laparoscopic surgery, *O* opening surgery

### Diagnostic performance of dual-tracer-guided SLN biopsy

**Fig. 2:**
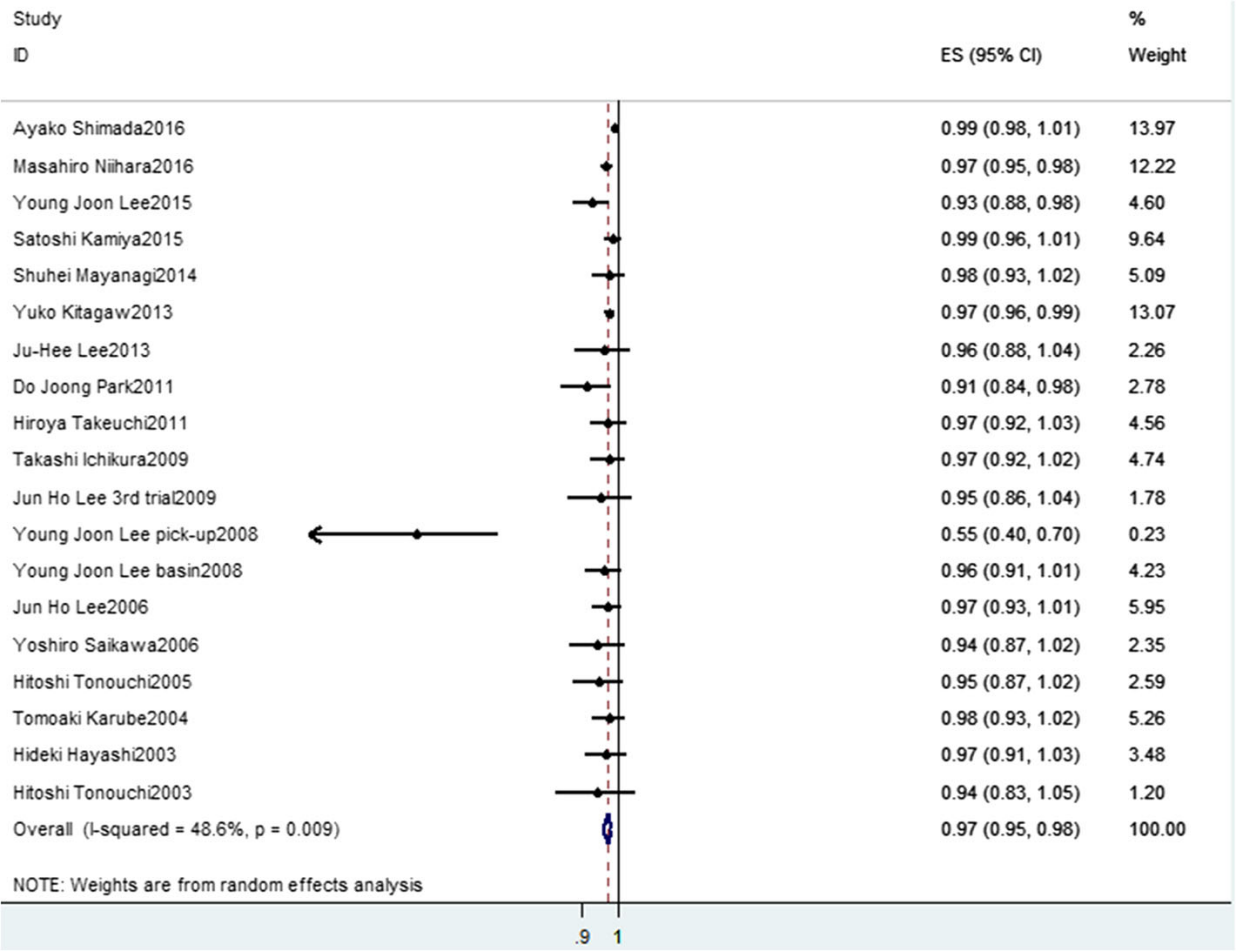
The pooled identification rate

**Fig. 3:**
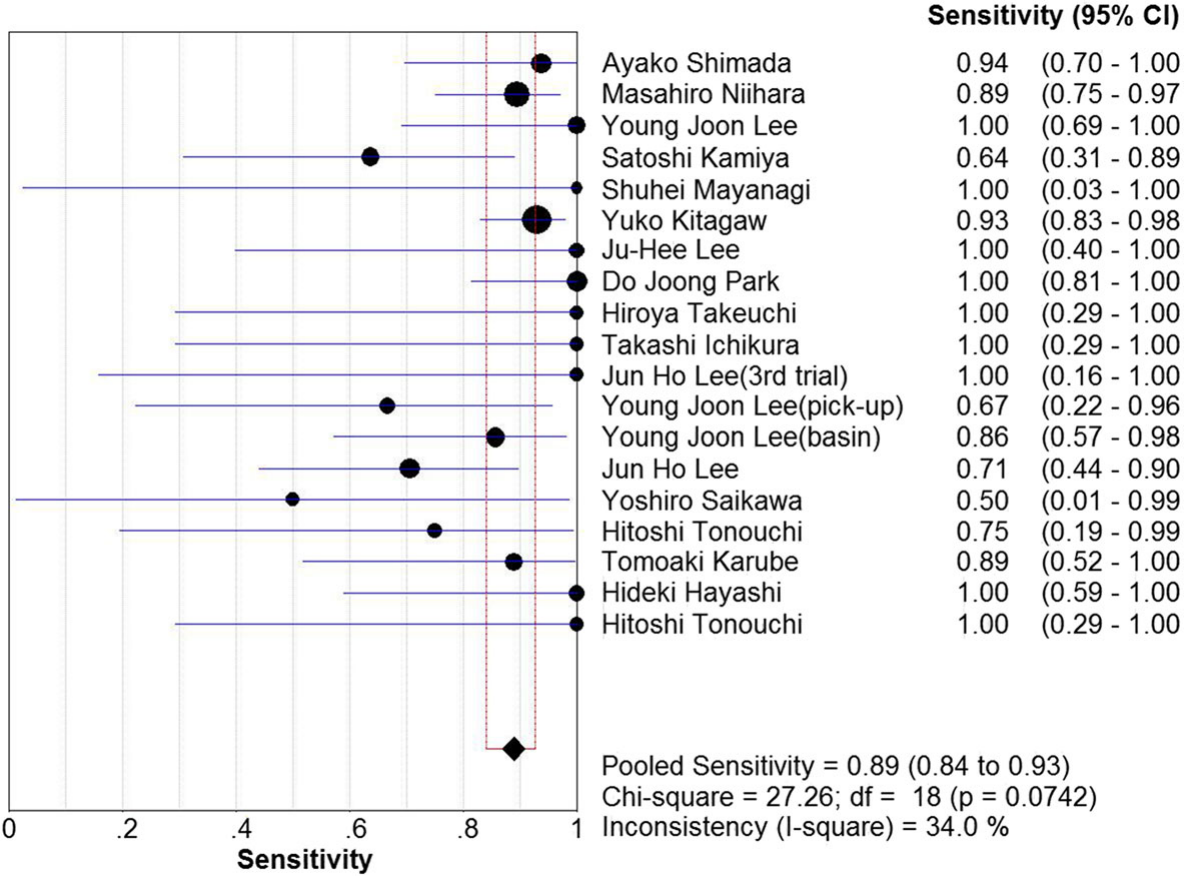
The pooled sensitivity

**Fig. 4:**
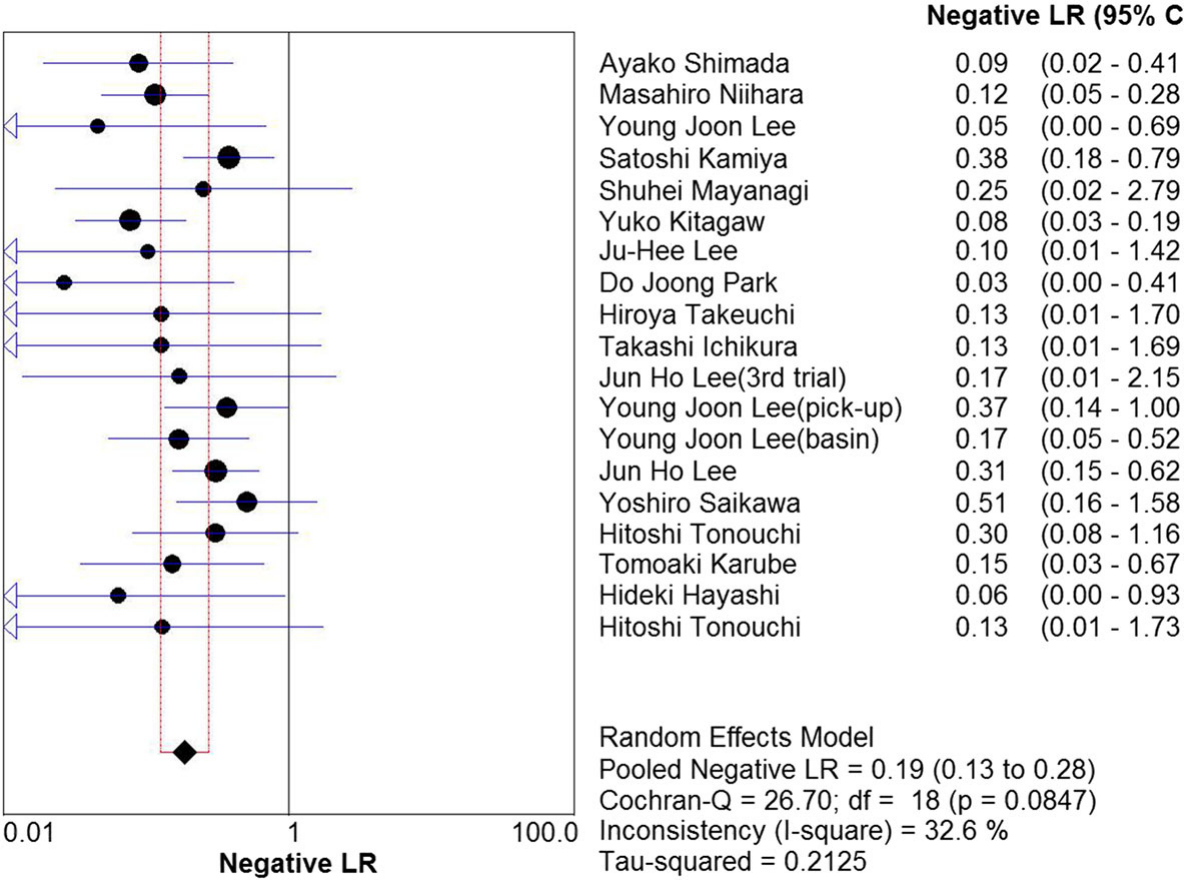
The pooled negative likelihood ratio. *LR* likelihood ratio

**Fig. 5:**
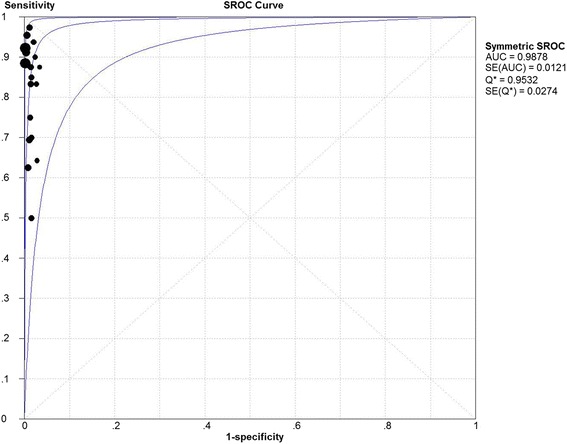
Summary receiver operator characteristic *curve*. *SROC* summary receiver operator characteristic, *AUC* the area under the *curve*

The pooled dual-tracer-guided SLN biopsy identification rate, sensitivity, and negative LR were 0.97 (95% confidence interval (CI), 0.95–0.98, *I*_2_ = 48.6%, Fig. 2), 0.89 (95%CI, 0.84–0.93, *I*_2_ = 34.0%, Fig. 3), and 0.19 (95%CI, 0.13–0.28, *I*_2_ = 32.6%, Fig. 4), respectively. Mild heterogeneities were found between studies with respect to identification rate, sensitivity, and negative LR according to the *I*_2_ value and *P* value. Spearman’s correlation coefficient was −0.301 (*P* = 0.21), indicating no threshold effect. The SROC curve is shown in Fig. 5; the AUC was 0.988 and Q* = 0.953, indicating the excellent effectiveness of the diagnostic technique.

### Subgroup analyses

The results of the subgroup analyses are shown in Table 3.

**Table 3:** Results of subgroup analyses

	Number of studies	Identification rate (95% CI)	*P*	Sensitivity (95% CI)	*P*
Preoperative T stage					
cT1	12	0.979 (0.923–1.035)	0.00	0.861 (0.786–0.917)	0.22
cT2	2	0.963 (0.751–1.176)	0.00	0.943 (0.808–0.993)	0.51
Number of SLNs					
<5	11	–	–	0.839 (0.760–0.900)	0.07
≥5	8	–	–	0.944 (0.882–0.979)	0.84
Method of SLN biopsy					
Pick up	13	0.973 (0.959–0.988)	0.01	0.869 (0.810–0.915)	0.11
Basin dissection	6	0.949 (0.925–0.974)	0.66	0.959 (0.860–0.995)	0.39
Type of dual tracers					
^99m^Tc tin colloid + isosulfan blue	3	0.971 (0.960–0.982)	0.77	0.884 (0.810–0.937)	0.07
^99m^Tc tin colloid + ICG	3	0.889 (0.779–1.000)	<.001	0.826 (0.612–0.950)	0.34
^99m^Tc tin colloid + patent blue violet	5	0.962 (0.933–0.992)	0.93	0.880 (0.688–0.975)	0.31
^99m^Tc-HSA + ICG	2	0.932 (0.890–0.977)	0.62	1.00 (0.735–1.000)	>0.99
^99m^Tc-antimony sulfur colloid + ICG	2	0.932 (0.882–0.985)	0.38	1.00 (0.846–1.000)	>0.99
Time performing SLN biopsy after dye injection					
≥15 min	9	0.973 (0.958–0.989)	0.10	0.952 (0.898–0.982)	0.71
<15 min	10	0.962 (0.939–0.986)	0.03	0.810 (0.719–0.882)	0.33
Method for intraoperative histological evaluation of SLN					
HE	7	–	–	0.908 (0.849–0.950)	0.11
HE + IHC	8	–	–	0.864 (0.750–0.940)	0.08
Methods of surgery					
Opening surgery	7	0.976 (0.965–0.988)	>.99	0.867 (0.786–0.925)	0.06
Laparoscopic surgery	10	0.939 (0.907–0.972)	0.04	0.909 (0.813–0.969)	0.13
Country					
Japan	12	0.980 (0.973–0.988)	0.54	0.896 (0.837–0.939)	0.31
Not in Japan	7	0.934 (0.891–0.978)	0.01	0.873 (0.773–0.940)	0.03
Year of studies					
≥2013	7	0.976 (0.963–0.990)	0.06	0.905 (0.843–0.949)	0.15
<2013	12	0.955 (0.931–0.980)	0.07	0.864 (0.774–0.928)	0.11

*SLN* sentinel lymph node, *P P* value of heterogeneity, *CI* confidence interval, *ICG* indocyanine green, ^*99m*^ *Tc-HSA* T^99m^c-human serum albumin, *HE* hematoxylin eosin, *IHC* immunohistochemistry

When considering the preoperative T stage, the pooled identification rate of the cT1 subgroup was a little higher than that of the cT2 subgroup, but there were significant heterogeneities in the pooled identification rate of both subgroups (*P* = 0.00). And the pooled sensitivity of the cT1 subgroup was lower than that of the cT2 subgroup (86.1 vs 94.3%).

When considering the type of dual tracer, the pooled sensitivity of the ^99m^Tc-human serum albumin and ICG subgroup and the ^99m^Tc-antimony sulfur colloid and ICG subgroup were the highest in five subgroups (100%).

When considering the time performing SLN biopsy after dye injection, the pooled identification rate and sensitivity of the ≥15 min subgroup was much higher than that of the <15 min subgroup (97.3 vs 96.2%, 95.2 vs 81.0%).

When considering the number of SLNs, the pooled sensitivity of the ≥5 subgroup was much higher than that of the <5 subgroup (94.4 vs 83.9%).

When considering the methods of SLN biopsy, the pooled sensitivity of the basin dissection subgroup was much higher than that of the pick-up subgroup (95.9 vs 86.9%). However, the pooled identification rate of the basin dissection subgroup was a little lower than that of the pick-up subgroup (94.9 vs 97.3%). And there were significant heterogeneities in the pooled identification rate of the pick-up subgroup (*P* = 0.01).

When considering the methods for the intraoperative histological evaluation of SLN, the pooled sensitivity of hematoxylin eosin (HE) was higher than that of the HE and immunohistochemistry (IHC) subgroup (90.8 vs 86.4%).

When considering the methods of surgery, the pooled identification rate of the opening surgery was a little higher than that of the laparoscopic subgroup (97.6 vs 93.9%). However, the pooled sensitivity of the laparoscopic surgery was a little higher than that of the opening surgery subgroup (90.9 vs 86.7%).

When considering the countries where researches were conducted, the pooled identification rate and sensitivity of the Japan subgroup was a little higher than that of the not in Japan subgroup (98.0 vs 93.4%, 89.6 vs 87.3%).

When considering the publication year, the pooled identification rate and sensitivity of the ≥2013 subgroup was higher than that of the <2013 subgroup (97.6 vs 95.5%, 90.5 vs 86.4%).

### Meta-regression analysis and publication bias

**Fig. 6:**
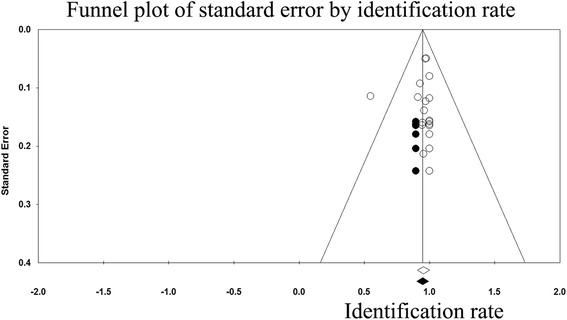
Funnel plot of the identification rate

**Fig. 7:**
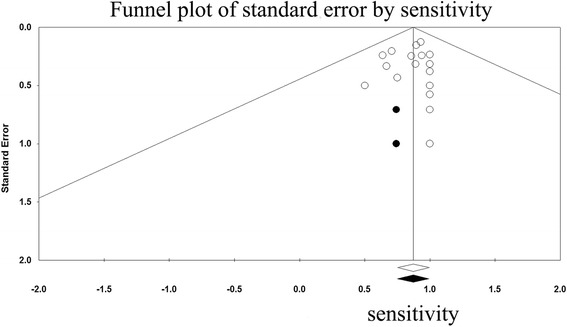
Funnel plot of sensitivity

No significant factors were found for the observed heterogeneity of sensitivity by the meta-regression analysis (Additional file 1: Table S3 illustrates the results of meta-regression analysis). With respect to the identification rate and sensitivity, funnel plots were generated to assess the publication bias of the included studies and the results suggested minimal bias (Fig. 6 and Fig. 7). These results were confirmed by Egger’s test, with *P* = 0.79 and 0.95, respectively.

## Discussion

To the best of our knowledge, this is the first meta-analysis on the detection rate and diagnostic performance of SLN biopsy guided by dual tracers in cT1-2N0M0 gastric cancer.

Our meta-analysis showed that the use of dual tracer in SLN mapping got a high pooled identification rate and sensitivity (97.0 and 89.0%). As reported by previous meta-analysis, radioactive isotope alone got a pooled identification rate and sensitivity of 92.1 and 76.4% while dye alone were 92.1 and 72.7% [32]. So, it seems that the diagnostic performance of dual tracer is the most excellent. The dye-guided method has the merits of safety, cheapness, and convenient injecting procedure and the ability of detecting not only lymph nodes but also lymphatic vessels. However, it is associated with a lower identification rate due to the rapid transit of the dye after injection and the blind sites in dense tissue [33]. On the other hand, the radiopharmaceutical-guided method has several advantages such as the objectivity of detecting SLN by quantitative evaluation of sentinel node radioactivity even in thick adipose tissue and suitability for laparoscopic surgery because of its longer deposition in lymph node than dye. But it also has several disadvantages such as high radioactivity interference at the primary injection site when detecting nearby SLNs and disability of visualizing the lymphatic vessels [34]. Thus, the combination of two kinds of tracer may be more reliable.

We performed subgroup analysis in terms of preoperative T stage. However, our results showed that the sensitivity of the cT1 subgroup was lower than that of the cT2 subgroup. This was different with the results of previous studies [32]. We could not find any explanations for this result: we reviewed the studies of cT1 subgroup and found most of the false-negative cases invaded muscularis propria according to the results of pathology detection. For instance, in the study by Niihara et al. which was classified as the cT1 subgroup, although there were three false-negative cases, the result of pathologic detection showed that two of them were pathologically T2 stage [15]. And in another cT1 study by Lee which was published in the year 2006, although there were five false-negative cases, the result of pathologic detection showed that four of them were pathologically T2 stage [27]. When tumors invade muscularis propria, the lymphatic drainage may be obstructed or altered and novel lymphangiogenesis makes lymphatic drainage more complicated. This may explain the reason why the sensitivity of SLN biopsy becomes lower in pT2 stage. Our results demonstrate that the accurate T stage before operation is important to optimize SLN biopsy and the clinical application of SLN biopsy should be limited to the patients of cT1N0M0 gastric cancer.

Because there were no uniform types of dual tracers in the included studies, we tried to figure out the effect of different types of dual tracers on the sensitivity and negative LR. The subgroup analysis showed that the combination of ^99m^Tc-human serum albumin and ICG and the combination of ^99m^Tc-antimony sulfur colloid and ICG had the highest sensitivity and satisfactory identification rate. The following points may be the reasons: firstly, both radioisotope ^99m^Tc-human serum albumin and ^99m^Tc-antimony sulfur colloid have a small particle size (≦200 nm) and have the advantages of migrating faster than ^99m^Tc tin colloid, being easy in predicting the proper time to probe, being able to be injected simultaneously with the dye, and being very stable. In addition, the tracer ^99m^Tc-human serum albumin has the merits of being biodegradable and reduced potential for allergic reactions [35, 36]. Secondly, the dye ICG was also reported to have a high detection rate and sensitivity. It has the merits of cheapness, convenience, being able to detect bright sentinel nodes in dense fat, signal stability, and clear visualization of lymph nodes and lymphatic canals. Besides, the recent technology such as HyperEye Medical System can simultaneously detect color and near-infrared rays of ICG and can be used under room light [37, 38]. However, the strength of this subgroup analysis is limited by its small sample size, and we hope that more relevant studies will be carried out in the future for a more precise evaluation.

We found that performing SLN biopsy ≥15 min after dye injection had a much higher identification rate and sensitivity than those of <15 min group. This result suggests that it is very important to control the time performing SLN biopsy because there will not be sufficient visualization of SLNs and lymphatic basins if researchers do biopsy in a hurry. However, the dye may diffuse and saturate the tissues which will make mapping difficult if it takes more than 20 min after dye injection [39, 40]. As for radioactive tracer, ^99m^Tc-tin colloid has been reported to be injected ranging from 2 h to the day before surgery while ^99m^Tc-human serum albumin is usually injected less than 30 min before surgery due to its small particle size [20].

In the meta-analysis by Ryu et al., sensitivity was found to depend significantly on the number of SLNs that were harvested [41]. Our result is consistent with the conclusion of this previous meta-analysis, as the pooled sensitivity for SLN number ≥5 group showed a significantly higher sensitivity than did the SLN number <5 group. These results can be explained by the complicated lymphatic drainage system of the stomach. Thus, as many SLNs as possible are necessary in the clinical application of SLN biopsy in gastric cancer.

Two methods to retrieve SLNs have been reported: the pick-up method and the basin dissection method. The gastric lymphatic basins were divided into five different directions along the main arteries. The dissected lymphatic basins contain lymph nodes and lymphatic vessels that are hot or stained with dye [20, 42]. Our subgroup analysis found that the basin subgroup had a much higher sensitivity than those of the pick-up group. This factor may be explained by the following reasons: when the lymphatic duct was dyed with invisible SLNs, basin dissection may solve this problem. And when SLNs are undetectable as the hot areas of them are close to the primary tumor, basin dissection may decrease false-negative rate. Moreover, a previous study also showed that more SLNs can be found using basin dissection than the pick-up method [25].

Our subgroup analysis showed that the sensitivity of the HE and IHC combined intraoperative pathologic detection method was lower than that of the HE alone group. This result is different from the result of a previous meta-analysis by Wang et al., who found the sensitivity of SLN biopsy by HE and IHC to be higher than that by HE alone (80.8 vs 73.7%) [32]. And the inverse result is explained by the following reasons: in the study by Kitagawa et al. which was classified as the HE subgroup, it was reported in the final result that only four false-negative cases were observed. However, it was also reported in the paper that this final result excluded nine patients who showed sentinel nodes negative in intraoperative examinations but positive in postoperative examinations of permanent tissue sections [20]. When it takes the sensitivity of intraoperative pathologic detection by frozen tissue sections into account, the pooled sensitivity of HE alone is 84.5%. And this is lower than the HE and IHC combined subgroup whose pooled sensitivity is 86.4%. Therefore, it is very important to improve the diagnostic accuracy of intraoperative pathologic examinations of frozen tissue sections. Except for combination of the HE and IHC method, multistep level sections, reverse transcription polymerase chain reaction (RT-PCR), and the one-step nucleic acid amplification assay (OSNA) have all been developed to improve the sensitivity of intraoperative pathologic diagnosis and detect SLN micrometastases. Moreover, many of these novel methods can be completed in 30 min which is convenient for intraoperative application [43].

Our subgroup analysis showed that the laparoscopic SLN biopsy got lower identification rate than the opening SLN biopsy. And this result may be due to the limited operation space and insufficient experience of surgeon when conducting laparoscopic SLN biopsy [44]. Despite these shortcomings, our subgroup analysis demonstrated that the sensitivity of the laparoscopic SLN biopsy was higher than the opening SLN biopsy. This may be explained by the more detailed observation with visual magnification during laparoscopic SLN biopsy [45]. Moreover, it was reported by previous study that laparoscopic surgery could decrease operative blood loss and hospital stay time as well as increased postoperative quality of life in gastric cancer patients compared with opening surgery [46]. We can anticipate that the laparoscopic SLN biopsy will have more and more advantages over opening SLN biopsy with the development of laparoscopic instruments as well as the improvement of experience of surgeons.

We assumed that the experience of surgeons and the application of dual-tracer-guided SLN biopsy would have improved with time. Thus, we set the year 2013 as the cut-off point to differentiate later and earlier studies. Improved identification rate and sensitivity were seen in the ≥2013 year subgroup. It was reported that sensitivity ranging from 90 to 95% was recommended for the feasibility of SLN biopsy clinical application in breast cancer [47]. As gastric cancer responds less to systemic adjuvant therapy or radiotherapy than breast cancer, some investigators argued that sensitivity of SLN biopsy should be higher for clinical application in gastric cancer than breast cancer. Therefore, although sensitivity of dual-tracer-guided SLN biopsy was significantly higher in the later studies than the earlier studies, the diagnostic performance of it in gastric cancer still need to be improved before finally putting into clinical application. Besides, lack of the approved area for injection of radioactive tracer and special instruments in the general hospitals is also an obstacle for the clinical application of this technique. Therefore, the single dye ICG-guided SLN biopsy has also been developed as a promising technique for gastric cancer recently. With the novel ICG fluorescence systems such as the D-light P system, the technique can be used in the room light and the laparoscopic surgery. It was reported in the small-scale studies that the identification rate and sensitivity of the technique could reach to 100% [37, 48, 49]. However, a large-scale multicenter clinical trial by the Japan Clinical Oncology Group reported that the detection rate of green nodes was 97%. However, the rate of false-negative was 46.4% which was surprisingly high [50]. Thus, further large-scale trials are needed to evaluate the clinical efficacy of this single dye method.

There were several limitations in our meta-analysis. First, the sample sizes of most of the included studies were small which may cause bias. Second, tumor size, pathologic type of tumor, and standard of preoperative clinical tumor stage between studies were not uniform, and all of which will affect the diagnostic performance of SLN biopsy. Moreover, all the involved studies are conducted in Japan and South Korea, and the performance of it in other countries is uncertain.

## Conclusions

The use of dual tracer is excellent and promising in SLN biopsy in gastric cancer. The accurate T stage before operation is important to optimize SLN biopsy, and the clinical application of SLN biopsy should be limited to the patients of cT1N0M0 gastric cancer. Our subgroup analysis showed that the combination of ^99m^Tc-human serum albumin and ICG as well as the combination of ^99m^Tc-antimony sulfur colloid and ICG may be the optimal combination. However, it seems not justified to put this technique into routine clinical application recently. Performing SLN biopsy ranging from 15 to 20 min after dye injection, SLN ≥5, basin dissection, and laparoscopic surgery can enhance the diagnostic value of this technique. Besides, it is very important to improve the diagnostic accuracy of intraoperative pathologic examinations of frozen tissue sections. Finally, we hope that more and more multicenter prospective clinical studies in different countries will be conducted to confirm the standard procedures and oncologic outcomes of this technique in the future.

## Additional File

Additional file 1: Table S1. Technical characteristics of the included studies. **Table S2.** Technical details and primary outcomes of the included studies. **Table S3.** Meta-regression analysis of sensitivity.

## Abbreviations

AUC: Area under the curve
CI: Confidence interval
FN: False negative
HE: Hematoxylin eosin
ICG: Indocyanine green
IHC: Immunohistochemistry
LR: Likelihood ratio
OSNA: One-step nucleic acid amplification
RT-PCR: Reverse transcription polymerase chain reaction
SLN: Sentinel lymph node
SROC: Summary receiver operator characteristic
TN: True negative
TP: True positive

## References

[1] Song M, Lee H-W, Kang D (2015). Epidemiology and screening of gastric cancer in Korea. J Kor Med Assoc.

[2] Inoue M, Tsugane S (2005). Epidemiology of gastric cancer in Japan. Postgrad Med J.

[3] Japanese Gastric Cancer Association. Japanese gastric cancer treatment guidelines 2014 (ver. 4). Gastric Cancer. 2017;20:1-19. 10.1007/s10120-016-0622-4 PMC5215069 27342689

[4] Ilhan E, Demir U, Alemdar A, Ureyen O, Eryavuz Y, Mihmanli M (2016). Management of high-output chylous ascites after D2-lymphadenectomy in patients with gastric cancer: a multi-center study. J Gastrointest Oncol.

[5] Takiguchi N, Takahashi M, Ikeda M, Inagawa S, Ueda S, Nobuoka T, Ota M, Iwasaki Y, Uchida N, Kodera Y, Nakada K (2015). Long-term quality-of-life comparison of total gastrectomy and proximal gastrectomy by Postgastrectomy Syndrome Assessment Scale (PGSAS-45): a nationwide multi-institutional study. Gastric Cancer.

[6] Park JY, Kim YW, Ryu KW, Nam BH, Lee YJ, Jeong SH, Park JH, Hur H, Han SU, Min JS (2016). Assessment of laparoscopic stomach preserving surgery with sentinel basin dissection versus standard gastrectomy with lymphadenectomy in early gastric cancer—a multicenter randomized phase III clinical trial (SENORITA trial) protocol. BMC Cancer.

[7] Goto O, Takeuchi H, Kawakubo H, Sasaki M, Matsuda T, Matsuda S, Kigasawa Y, Kadota Y, Fujimoto A, Ochiai Y (2015). First case of non-exposed endoscopic wall-inversion surgery with sentinel node basin dissection for early gastric cancer. Gastric Cancer.

[8] Takayama T, Matsumoto S, Wakatsuki K, Tanaka T, Migita K, Ito M, Nakajima Y (2014). A novel laparoscopic procedure for treating proximal early gastric cancer: laparoscopy-assisted pylorus-preserving nearly total gastrectomy. Surg Today.

[9] Straver ME, Meijnen P, van Tienhoven G, van de Velde CJ, Mansel RE, Bogaerts J, Duez N, Cataliotti L, Klinkenbijl JH, Westenberg HA (2010). Sentinel node identification rate and nodal involvement in the EORTC 10981-22023 AMAROS trial. Ann Surg Oncol.

[10] Lee SE, Lee JH, Ryu KW, Cho SJ, Lee JY, Kim CG, Choi IJ, Kook MC, Nam BH, Park SR (2009). Sentinel node mapping and skip metastases in patients with early gastric cancer. Ann Surg Oncol.

[11] Tani T, Sonoda H, Tani M (2016). Sentinel lymph node navigation surgery for gastric cancer: does it really benefit the patient?. World J Gastroenterol.

[12] Whiting PF, Rutjes AW, Westwood ME, Mallett S, Deeks JJ, Reitsma JB, Leeflang MM, Sterne JA, Bossuyt PM (2011). QUADAS-2: a revised tool for the quality assessment of diagnostic accuracy studies. Ann Intern Med.

[13] Rosman AS, Korsten MA (2007). Application of summary receiver operating characteristics (sROC) analysis to diagnostic clinical testing. Adv Med Sci.

[14] Shimada A, Takeuchi H, Ono T, Kamiya S, Fukuda K, Nakamura R, Takahashi T, Wada N, Kawakubo H, Saikawa Y (2016). Pylorus-preserving surgery based on the sentinel node concept in early gastric cancer. Ann Surg Oncol.

[15] Niihara M, Takeuchi H, Nakahara T, Saikawa Y, Takahashi T, Wada N, Mukai M, Kitagawa Y (2016). Sentinel lymph node mapping for 385 gastric cancer patients. J Surg Res.

[16] Lee YJ, Jeong SH, Hur H, Han S-U, Min JS, An JY, Hyung WJ, Cho GS, Jeong GA, Jeong O, et al. Prospective multicenter feasibility study of laparoscopic sentinel basin dissection for organ preserving surgery in gastric cancer quality control study for surgical standardization prior to phase III trial. Med. 2015;94:e1894. 10.1097/MD.0000000000001894 PMC4985421 26512607

[17] Kamiya S, Takeuchi H, Nakahara T, Niihara M, Nakamura R, Takahashi T, Wada N, Kawakubo H, Saikawa Y, Omori T (2016). Auxiliary diagnosis of lymph node metastasis in early gastric cancer using quantitative evaluation of sentinel node radioactivity. Gastric Cancer.

[18] Mayanagi S, Takeuchi H, Kamiya S, Niihara M, Nakamura R, Takahashi T, Wada N, Kawakubo H, Saikawa Y, Omori T (2014). Suitability of sentinel node mapping as an index of metastasis in early gastric cancer following endoscopic resection. Ann Surg Oncol.

[19] Lee J-H, Park DJ, Kim YH, Shin C-M, Lee HS, Kim H-H (2013). Clinical implementations of preoperative computed tomography lymphography in gastric cancer: a comparison with dual tracer methods in sentinel node navigation surgery. Ann Surg Oncol.

[20] Kitagawa Y, Takeuchi H, Takagi Y, Natsugoe S, Terashima M, Murakami N, Fujimura T, Tsujimoto H, Hayashi H, Yoshimizu N (2013). Sentinel node mapping for gastric cancer: a prospective multicenter trial in Japan. J Clin Oncol.

[21] Park DJ, Kim H-H, Park YS, Lee HS, Lee WW, Lee H-J, Yang H-K (2011). Simultaneous indocyanine green and Tc-99m-antimony sulfur colloid-guided laparoscopic sentinel basin dissection for gastric cancer. Ann Surg Oncol.

[22] Takeuchi H, Oyama T, Kamiya S, Nakamura R, Takahashi T, Wada N, Saikawa Y, Kitagawa Y (2011). Laparoscopy-assisted proximal gastrectomy with sentinel node mapping for early gastric cancer. World J Surg.

[23] Ichikura T, Sugasawa H, Sakamoto N, Yaguchi Y, Tsujimoto H, Ono S (2009). Limited gastrectomy with dissection of sentinel node stations for early gastric cancer with negative sentinel node biopsy. Ann Surg.

[24] Lee JH, Ryu KW, Nam BH, Kook MC, Cho SJ, Lee JY, Kim CG, Choi IJ, Park SR, Kim YW (2009). Factors associated with detection failure and false-negative sentinel node biopsy findings in gastric cancer: results of prospective single center trials. J Surg Oncol.

[25] Lee YJ, Ha WS, Park ST, Choi SK, Hong SC, Park JW (2008). Which biopsy method is more suitable between a basin dissection and pick-up biopsy for sentinel nodes in laparoscopic sentinel-node navigation surgery (LSNNS) for gastric cancer?. J Laparoendosc Adv Surg Tech A.

[26] Saikawa Y, Otani Y, Kitagawa Y, Yoshida M, Wada N, Kubota T, Kumai K, Sugino Y, Mukai M, Kameyama K (2006). Interim results of sentinel node biopsy during laparoscopic gastrectomy: possible role in function-preserving surgery for early cancer. World J Surg.

[27] Lee JH, Ryu KW, Kim CG, Kim SK, Lee JS, Kook MC, Choi IJ, Kim YW, Chang HJ, Bae JM (2006). Sentinel node biopsy using dye and isotope double tracers in early gastric cancer. Ann Surg Oncol.

[28] Karube T, Ochiai T, Shimada H, Nikaidou T, Hayashi H (2004). Detection of sentinel lymph nodes in gastric cancers based on immunohistochemical analysis of micrometastases. J Surg Oncol.

[29] Tonouchi H, Mohri Y, Tanaka K, Konishi N, Ohmori Y, Kobayashi M, Watanabe Y, Matsumura K, Takeda K, Kusunoki M (2003). Lymphatic mapping and sentinel node biopsy during laparoscopic gastrectomy for early cancer. Dig Surg.

[30] Hayashi H, Ochiai T, Mori M, Karube T, Suzuki T, Gunji Y, Hori S, Akutsu N, Matsubara H, Shimada H (2003). Sentinel lymph node mapping for gastric cancer using a dual procedure with dye- and gamma probe-guided techniques. J Am Coll Surg.

[31] Tonouchi H, Mohri Y, Tanaka K, Kobayashi M, Ohmori Y, Kusunoki M (2005). Laparoscopic lymphatic mapping and sentinel node biopsies for early-stage gastric cancer: the cause of false negativity. World J Surg.

[32] Wang Z, Dong ZY, Chen JQ, Liu JL (2012). Diagnostic value of sentinel lymph node biopsy in gastric cancer: a meta-analysis. Ann Surg Oncol.

[33] Kitagawa Y, Fujii H, Kumai K, Kubota T, Otani Y, Saikawa Y, Yoshida M, Kubo A, Kitajima M (2005). Recent advances in sentinel node navigation for gastric cancer: a paradigm shift of surgical management. J Surg Oncol.

[34] Yashiro M, Matsuoka T (2015). Sentinel node navigation surgery for gastric cancer: overview and perspective. World J Gastrointest Surg.

[35] Bedrosian I, Scheff AM, Mick R, Callans LS, Bucky LP, Spitz FR, Helsabeck C, Elder DE, Alavi A, Fraker DF, Czerniecki BJ (1999). 99mTc-human serum albumin: an effective radiotracer for identifying sentinel lymph nodes in melanoma. J Nucl Med.

[36] Takeuchi H, Kitagawa Y (2013). New sentinel node mapping technologies for early gastric cancer. Ann Surg Oncol.

[37] Kinami S, Oonishi T, Fujita J, Tomita Y, Funaki H, Fujita H, Nakano Y, Ueda N, Kosaka T (2016). Optimal settings and accuracy of indocyanine green fluorescence imaging for sentinel node biopsy in early gastric cancer. Oncol Lett.

[38] Yoshida M, Kubota K, Kuroda J, Ohta K, Nakamura T, Saito J, Kobayashi M, Sato T, Beck Y, Kitagawa Y, Kitajima M (2012). Indocyanine green injection for detecting sentinel nodes using color fluorescence camera in the laparoscopy-assisted gastrectomy. J Gastroenterol Hepatol.

[39] Lee CM, Park S, Park SH, Jung SW, Choe JW, Sul JY, Jang YJ, Mok YJ, Kim JH. Sentinel node mapping using a fluorescent dye and visible light during laparoscopic gastrectomy for early gastric cancer: result of a prospective study from a single institute. Ann Surg. 2017;265:766–773. 10.1097/SLA.0000000000001739 27058946

[40] How J, Gotlieb WH, Press JZ, Abitbol J, Pelmus M, Ferenczy A, Probst S, Gotlieb R, Brin S, Lau S (2015). Comparing indocyanine green, technetium, and blue dye for sentinel lymph node mapping in endometrial cancer. Gynecol Oncol.

[41] Ryu KW, Eom BW, Nam BH, Lee JH, Kook MC, Choi IJ, Kim YW (2011). Is the sentinel node biopsy clinically applicable for limited lymphadenectomy and modified gastric resection in gastric cancer? A meta-analysis of feasibility studies. J Surg Oncol.

[42] Kinami S, Fujimura T, Ojima E, Fushida S, Ojima T, Funaki H, Fujita H, Takamura H, Ninomiya I, Nishimura G (2008). PTD classification: proposal for a new classification of gastric cancer location based on physiological lymphatic flow. Int J Clin Oncol.

[43] Lianos GD, Hasemaki N, Vaggelis G, Karampa A, Anastasiadi Z, Lianou A, Papanikolaou S, Floras G, Bali CD, Lekkas E (2016). Sentinel node navigation in gastric cancer: new horizons for personalized minimally invasive surgical oncology?. Transl Gastroenterol Hepatol.

[44] Kataoka K, Katai H, Mizusawa J, Katayama H, Nakamura K, Morita S, Yoshikawa T, Ito S, Kinoshita T, Fukagawa T, Sasako M (2016). Non-randomized confirmatory trial of laparoscopy-assisted total gastrectomy and proximal gastrectomy with nodal dissection for clinical stage I gastric cancer: Japan Clinical Oncology Group Study JCOG1401. J Gastric Cancer.

[45] Lu W, Gao J, Yang J, Zhang Y, Lv W, Mu J, Dong P, Liu Y (2016). Long-term clinical outcomes of laparoscopy-assisted distal gastrectomy versus open distal gastrectomy for early gastric cancer: a comprehensive systematic review and meta-analysis of randomized control trials. Medicine (Baltimore).

[46] Li HZ, Chen JX, Zheng Y, Zhu XN (2016). laparoscopic-assisted versus open radical gastrectomy for resectable gastric cancer: systematic review, meta-analysis, and trial sequential analysis of randomized controlled trials. J Surg Oncol.

[47] Kim T, Giuliano AE, Lyman GH (2006). Lymphatic mapping and sentinel lymph node biopsy in early-stage breast carcinoma: a metaanalysis. Cancer.

[48] Tummers QR, Boogerd LS, de Steur WO, Verbeek FP, Boonstra MC, Handgraaf HJ, Frangioni JV, van de Velde CJ, Hartgrink HH, Vahrmeijer AL (2016). Near-infrared fluorescence sentinel lymph node detection in gastric cancer: a pilot study. World J Gastroenterol.

[49] Takahashi N, Nimura H, Fujita T, Yamashita S, Mitsumori N, Yanaga K (2016). Quantitative assessment of visual estimation of the infrared indocyanine green imaging of lymph nodes retrieved at sentinel node navigation surgery for gastric cancer. BMC Surg.

[50] Miyashiro I, Hiratsuka M, Sasako M, Sano T, Mizusawa J, Nakamura K, Nashimoto A, Tsuburaya A, Fukushima N (2014). High false-negative proportion of intraoperative histological examination as a serious problem for clinical application of sentinel node biopsy for early gastric cancer: final results of the Japan Clinical Oncology Group multicenter trial JCOG0302. Gastric Cancer.

